# Walnut Polyphenol Extract Attenuates Immunotoxicity Induced by 4-Pentylphenol and 3-methyl-4-nitrophenol in Murine Splenic Lymphocyte

**DOI:** 10.3390/nu8050287

**Published:** 2016-05-12

**Authors:** Lubing Yang, Sihui Ma, Yu Han, Yuhan Wang, Yan Guo, Qiang Weng, Meiyu Xu

**Affiliations:** 1 Collage of Biological Science and Technology, Beijing Forestry University, Beijing 100083, China; yanglubingo@163.com (L.Y.); masihuikindle@163.com (S.M.); rebeccahan@bjfu.edu.cn (Y.H.); wwangyuhann@163.com (Y.W.); qiangweng@bjfu.edu.cn (Q.W.); 2 Beijing Key Laboratory of Forest Food Processing and Safety, Beijing Forestry University, Beijing 100083, China; 3 College of Basic Medicine, Changchun University of Traditional Chinese Medicine, Changchun 130117, China; ccguoyan@163.com

**Keywords:** 3-methyl-4-nitrophenol, 3-methyl-4-nitrophenol, walnut polyphenol extract, attenuates, immunotoxicity, splenic lymphocyte

## Abstract

4-pentylphenol (PP) and 3-methyl-4-nitrophenol (PNMC), two important components of vehicle emissions, have been shown to confer toxicity in splenocytes. Certain natural products, such as those derived from walnuts, exhibit a range of antioxidative, antitumor, and anti-inflammatory properties. Here, we investigated the effects of walnut polyphenol extract (WPE) on immunotoxicity induced by PP and PNMC in murine splenic lymphocytes. Treatment with WPE was shown to significantly enhance proliferation of splenocytes exposed to PP or PNMC, characterized by increases in the percentages of splenic T lymphocytes (CD3+ T cells) and T cell subsets (CD4+ and CD8+ T cells), as well as the production of T cell-related cytokines and granzymes (interleukin-2, interleukin-4, and granzyme-B) in cells exposed to PP or PNMC. These effects were associated with a decrease in oxidative stress, as evidenced by changes in OH, SOD, GSH-Px, and MDA levels. The total phenolic content of WPE was 34,800 ± 200 mg gallic acid equivalents/100 g, consisting of at least 16 unique phenols, including ellagitannins, quercetin, valoneic acid dilactone, and gallic acid. Taken together, these results suggest that walnut polyphenols significantly attenuated PP and PNMC-mediated immunotoxicity and improved immune function by inhibiting oxidative stress.

## 1. Introduction

Vehicle emissions have been shown to significantly inhibit endocrine [1,2,3], reproductive [4,5,6,7], and immune system [8,9] function through a variety of different mechanisms. Diesel exhaust particles confer toxicity in human neutrophil granulocytes, rat alveolar macrophages, and murine RAW 264.7 macrophages [10,11], and suppress cytokine release [12], significantly diminishing immune function [13,14], while gasoline exhaust particles have also been shown to inhibit T-cell proliferation [15]. Among the bioactive compounds found in vehicle emissions, two chemicals, 4-pentylphenol (PP) and 3-methyl-4-nitrophenol (PNMC), have been shown to significantly inhibit splenocyte viability and cytokine production, indicating immunotoxicity of the two vehicle exhaust [16,17,18].

Dietary polyphenols have been shown to confer protective activity against a variety of toxins. One such polyphenol, proanthocyanidin, significantly attenuated cadmium-induced renal damage in mice [19], inhibited neurological impairments caused by lead exposure in rats [20], and prevented nodularin-mediated lymphocyte toxicity in *Carassius auratus* [21]. Similarly, another polyphenol, quercetin, protected against male reproductive toxicity caused by PNMC in germ cells of embryonic chickens and mice [22,23,24], as well as inhibited atrazine-induced damage in the liver, kidney, brain, and heart of adult Wistar rats [25]. However, despite these preliminary observations, little is known about the role of these compounds in preventing the immunotoxicity caused by PP or PNMC.

Walnuts (*Juglans regia* L.) are not only an excellent source of essential unsaturated fatty acids (linoleic and α-linolenic acids) but are also rich in polyphenols [26], ranking second in antioxidant content among 1113 different foods evaluated [27]. Beneficial properties associated with walnut extracts include antibacterial, anticancer, hepatoprotective, antidiabetic, anti-inflammatory, anti-depressive, and antioxidative activities [28]. Walnut polyphenols were shown to protect against CCl_4_-induced oxidative damage in rat liver, inflammation and cellular dysfunction in rat primary hippocampal neurons, amyloid beta protein-induced oxidative stress and cell death [29,30,31], and cisplatin-induced disruptions in motor and cognitive function [32]. However, despite these well-documented activities, the effect of walnut polyphenols on immune toxicity is unknown. Here, we investigated the potential protective effects of walnut polyphenol extract (WPE) on PP- and PNMC-induced immunotoxicity and evaluated the relationship between immunotoxicity and oxidative stress. Finally, we sought to identify the individual phenolic constituents contained within WPE.

## 2. Materials and Methods

### 2.1. Materials

Walnuts were obtained from the Jingpin Fruit Industry Co., Ltd (Hebei, China). Quercetin (purity ≥ 98%) was purchased from Tauto Biotech Co., Ltd. (Shanghai, China), and proanthocyanidin (purity ≥ 95%) from Jianfeng Natural Product R and D Co., Ltd. (Tianjin, China). PP was purchased from Sigma (St. Louis, MO, USA) and PNMC from TCI Chemicals (Tokyo, Japan). RPMI 1640 medium and phosphate-buffered saline (PBS, pH 7.4) were obtained from Mediatech (Manassas, VA, USA). Pharmingen Stain Buffer (BSA) was obtained from BD (Becton Dickinson, San Diego, CA, USA). 3-(4,5-dimethylthiazol-2-yl)-2,5-diphenyl tetrazolium bromide (MTT) was purchased from Sigma. ELISA kits for IL-2, IL-4, and Granzyme B were purchased from Cusabio Biotech (Wuhan, China). Assay kits of hydroxyl free radical (·OH), superoxide dismutase (SOD), glutathione peroxidase (GSH-Px), and malondialdehyde (MDA) were bought from the Nanjing Jiancheng Bioengineering Institute (Nanjing, China). LC–MS grade solvents were obtained from Honeywell Burdick and Jackson (Muskegon, MI, USA). All other commercial reagents were of analytical grade and were purchased from local commercial firms.

The following antibodies purchased from Biogen (San Diego, CA, USA) were used in the phenotypic analysis studies: fluorescein isothiocyanate (FITC)-labeled anti-mouse CD3 (IgG_2b_) to stain T-cells, FITC-anti-mouse CD4 (IgG_2b_) and FITC-anti-mouse CD8 (IgG_2b_) to stain T-cell subsets, and FITC-anti-mouse CD19 (IgG_2a_) to stain B-cells. FITC-labeled rat IgG_2a_ and IgG_2b_ were used as negative isotype controls.

### 2.2. Experimental Animals

Specific-pathogen-free Kunming mice (male, eight weeks of age) were purchased from the Military Academy of Medical Sciences Laboratory Animal Center (Beijing, China) to serve as the source of cells for use in all assays herein. The mice were housed in a pathogen-free facility maintained at a temperature of 23–25 °C and a relative humidity at 57%–60% with a 12-h light-dark cycle. All mice had *ad libitum* access to standard sterilized rodent chow and filtered water. All procedures here were carried out in accordance with the Policy on the Care and Use of Animals established by the Ethical Committee of the Beijing Forestry University and approved by the Department of Agriculture of Hebei Province, China (JNZF11/2007).

### 2.3. Preparation of WPE

The WPE was prepared by the method of Muthaiyah *et al.* [31]. In brief, walnuts (30 g) were frozen for 24 h; the shelled kernels were ground with a mechanical grinder and then immersed in 240 mL of 100 mM acetate buffer, pH 4.8/acetone (30:70, *v*/*v*) at 4 °C for 24 h. This process was repeated. The two extracts were combined and concentrated using a rotary evaporator under reduced pressure at 37 °C until the organic solvent was completely evaporated. The concentrated solution was extracted three times with 75 mL ethyl acetate. The three ethyl acetate extracts were combined and then evaporated to remove ethyl acetate. Powder of WPE was obtained by lyophilizing.

### 2.4. Preparation of Splenocytes

Based on the protocols of Benencia *et al.* [33], naïve mice were euthanized by cervical dislocation and its spleen was removed. Single cell suspensions were prepared by mincing and tapping spleen fragments on a stainless 200-mesh held in RPMI 1640 medium. Thereafter, erythrocytes present were lysed by incubating the cells in ammonium chloride (0.8%, *w*/*v*) solution on ice for 2 min. After centrifugation (380× *g*, 5 min), the pelleted cells were washed three times with RPMI-1640 and finally re-suspended in RPMI-1640 supplemented with 10% fetal bovine serum, 2 mM l-glutamine, 100 U penicillin/mL, and 100 μg streptomycin/mL (all products from Mediatech). Cell number and viability were determined using a hemocytometer and trypan blue dye. Cell viability always exceeded 95%.

### 2.5. Cell Viability Assay

Measurement of cell survival was assayed as previously described [34]. In brief, one hundred microliters of splenocyte suspension (5 × 10^6^ cell/mL) was aliquoted into each well of a 96-well flat-bottom microtiter plates. After 4 h incubation, 100 µL PP/PNMC (10^−4^ M final concentration in well, optimal dose as determined by preliminary experiments) alone or in combinations with WPE (0.01, 0.1, 1.0, 2.0, 3.0, 4.0, 5.0, and 10.0 μg/mL final concentration in well), Que (1.0 μg/mL final concentration in well), or PC (1.0 μg/mL final concentration in well) was added to designated wells. Cells treated with RPMI 1640 medium were used as positive control. The cells were then incubated at 37 °C in a humidified atmosphere with 5% CO_2_ for another 48 h. At the end of the exposure, 20 μL MTT solution (5 mg/mL) were then added into each well. The samples were incubated a further 4 h. The culture supernatant was carefully removed, and 200 μL DMSO was added to each well. The plate was shaken slightly for 20 min and the absorbance was determined at 570 nm using an ELISA plate reader (BioRad, Hercules, CA, USA). Cell viability (%) = (the absorbance of experiment group/the absorbance of control group) × 100%.

### 2.6. Cell Staining/Flow Cytometry for Phenotypic Analysis

Cell staining for phenotypic analysis was carried out as described previously [9,35]. In short, the treated spleen cells were harvested, washed with PBS, then diluted to 2.5 × 10^7^ cells/mL in PBS. Re-centrifuged the cells and then re-suspended in 50 µL Ab Block (BD Pharmingen Stain Buffer (BSA)) for 5 min at 4 °C. After blocking, the cells were added a given specific FITC-labeled antibody at 1 µg/mL (optimal dose as determined by preliminary experiments), then incubated for 30 min at 4 °C in the dark. After staining, the cells were washed three times with PBS and centrifugation. After the final washing, the cells were transferred to FACS tubes (in PBS) for phenotypic analysis using a BD FACS Calibur flow cytometer (Becton Dickinson, San Diego, CA, USA) equipped with FloJo software (Emerald Biotech, Hangzhou, China) for data analysis. Cells were excited with a 488 nm argon laser line and the fluorescence of FITC was analyzed on FL1 (530 nm), counting 10,000 events per sample. Splenocytes were electronically gated to exclude any residual platelets, red cells, or dead cell debris. The results were indicated as the percentage positive cells within a gate which was the same for both exposed and control splenocytes.

### 2.7. Measurement of Cytokine/Granzyme Production and Determination of ·OH, SOD, GSH-Px, and MDA Levels

For assessment of IL-2, IL-4, granzyme-B, SOD, GSH-Px, ·OH, and MDA levels, splenocytes was treated with the test reagents for 48 h at a density of 5 × 10^6^ cells/mL in 96-well plates. Culture supernatant was then collected and quantified following procedures by commercial ELISA kits. The lower detection limit of the kits was 3.9 pg IL-2/mL, 0.4 pg IL-4/mL, 3.1 pg Granzyme B/mL, 0.5 U SOD/mL, 0.5 U GSH-Px/mL, 0.04 U ·OH/mL, and 0.01 mmol MDA/mL. 

### 2.8. Determination of the Total Phenolic Content

The total phenolic content was determined by the Folin–Ciocalteu phenol reagent method prescribed by Wang *et al.* [36]. 200 μL of WPE was transferred to a 10 mL volumetric flask, to which 0.5 mL of Folin–Ciocalteu reagent was added. After 1 min, 1.5 mL of 20% (*w*/*v*) Na_2_CO_3_ was added and the volume was made up to 10 mL with distilled water. After 2 h incubation at 40 °C, the absorbance was measured at 760 nm in a 722 UV-VIS Spectrum Spectrophotometer (ShunYu Constant Scientific Instrument co., Ltd., Shanghai, China). The total phenolic content was calculated on the basis of the standard curve for gallic acid solutions and expressed as g of gallic acid equivalents (GAE)/100 g of sample.

### 2.9. LC-MS Analyses (HPLC-ESI-IT-TOF-MS)

LC–MS analysis was conducted as described previously [37,38] and with some modifications. LC–MS analysis was performed using a Shimadzu ESI-IT-TOF-MS instrument (Shimadzu, Tokyo, Japan) equipped with a HPLC system (SIL-20A HT autosampler, LC-20AD pump system, SDP-M20A photo diode array detector). The LC separation was performed using a C18 reverse-phase column (Shimpack XR-ODS column, 50 mm × 3.0 mm id × 2.2 μm; Shimadzu Scientific Instruments Inc., Columbia, MD, USA) and maintained at 30 °C with a solvent system comprising of 0.1% formic acid in H_2_O (A) and 0.1% formic acid in acetonitrile (B). Prior to the injection, the column was equilibrated for 5 min at initial conditions (5% B). Compounds were eluted into the ion source at a flow rate of 1 mL/min with a step gradient of 5%–95% B over 30 min, isocratic at 95% B over 2 min, and return to 5% B over 1 min. The injection volume was set to 10 μL. The heat block and curved desolvation line of were maintained at 200 °C. Nitrogen gas was used as nebulizer and drying gas with the flow rate set at 1.5 L/min. The ESI source voltage was set at 4.5 kV and the detector was set at 1.5 V. Ionization was performed using a conventional ESI source in negative ionization mode. Data was acquired from *m*/*z* 100–1000. Shimadzu’s LC-MS Solution software (Shimadzu Scientific Instruments Inc.) was used for system control and data analysis. Each compound was identified by comparison between retention time, compound spectra, as well as mass (*m*/*z*), with their counterparts in previous studies (please see reference in Table 1).

### 2.10. Statistical Analysis

All data were expressed as means ± SD. Statistical analyses were performed using one-way analysis of variance (ANOVA) followed by a *post hoc* test, Tukey’s test (as part of SPSS software package (IBM, Armonk, NY, USA)). Significance was accepted at a *p*-value < 0.05.

## 3. Results

### 3.1. WPE Attenuates Cytotoxicity in Splenocytes

**Figure 1:**
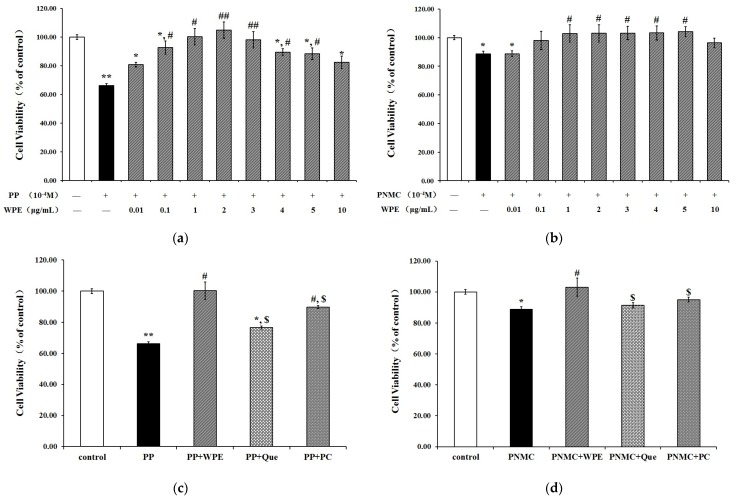
Effect of WPE on cytotoxicity in splenocytes exposed to PP/PNMC. Splenocytes were treated with (**A**) PP (10^−4^ M) or different concentrations (0.01, 0.1, 1.0, 2.0, 3.0, 4.0, 5.0, and 10.0 μg/mL) of WPE together with PP; (**B**) PNMC (10^−4^ M) or different concentrations (0.01, 0.1, 1.0, 2.0, 3.0, 4.0, 5.0, and 10.0 μg/mL) of WPE together with PNMC; (**C**) PP (10^−4^ M) or WPE (1.0 μg/mL), Que (1.0 μg/mL), PC (1.0 μg/mL) together with PP (**D**) PNMC (10^−4^ M) or WPE (1.0 μg/mL), Que (1.0 μg/mL), PC (1.0 μg/mL) together with PNMC for 48 h. Que, quercetin; PC, proanthocyanidin. Results shown are means ± SD of three separate experiments. * *p* < 0.05 or ** *p* < 0.01 *vs.* untreated control; ^#^ *p* < 0.05 or ^##^ *p* < 0.01 *vs.* PP or PNMC treatment; ^$^ *p* < 0.05 *vs.* WPE (1.0 μg/mL), together with PP (10^−4^ M) or PNMC (10^−4^ M) treatment.

Splenocyte cells exposed to PP or PNMC were examined by MTT assay to assess the effect of WPE on cell viability. PP and PNMC significantly decreased cell viability to 66% and 88%, respectively, relative to controls. These effects were significantly attenuated in cells treated with WPE (Figure 1A,B), which limited cytotoxicity in a concentration-dependent manner at concentrations of 0.01–1.0 μg/mL. Treatment of splenocytes with 1.0 μg/mL WPE increased cell viability from 66% to 100% and from 88% to 102% in PP- and PNMC-treated cells, respectively, relative to controls. As no additional benefits were seen at concentrations > 1.0 µg/mL WPE, this concentration was chosen for use in all subsequent experiments. Treatment with quercetin or proanthocyanidin (1 µg/mL) increased cell viability (Figure 1C,D), but the protective effects of the two compounds were weaker than those of WPE. These data showed that WPE protected against PP- and PNMC-induced cytotoxicity in splenocytes.

### 3.2. WPE Inhibited Decreases in Splenic T Cell Sub-Populations

**Figure 2:**
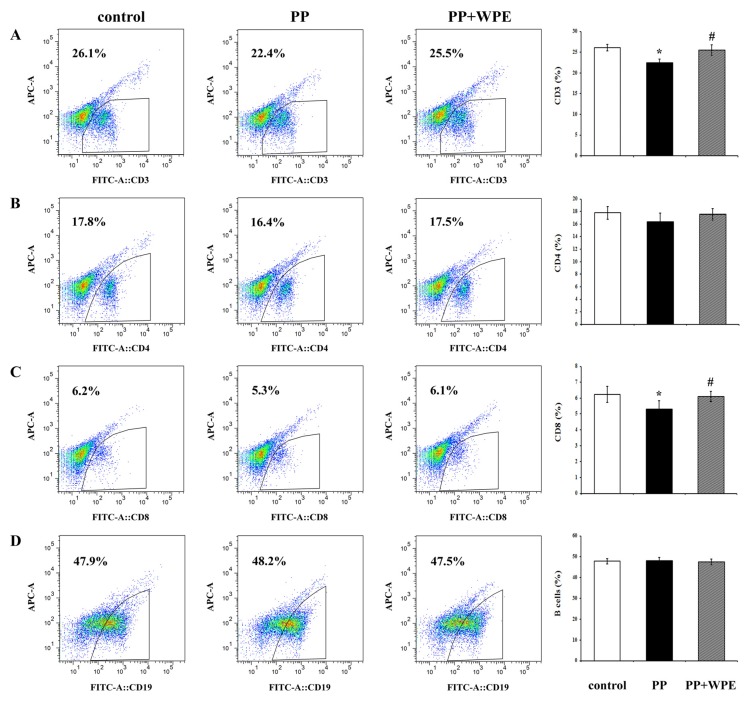
Percentages of various lymphocyte cell types as determined using flow cytometric analysis. (**A**) CD3^+^ T-cells; (**B**) CD4^+^ T-cells; (**C**) CD8^+^ T-cells; and (**D**) B-cells in cells exposed to medium only (control), PP (10^−4^ M), or WPE (1.0 μg/mL), together with PP for 48 h. Results shown are means ± SD of three separate experiments. * *p* < 0.05 *vs.* untreated control; ^#^ *p* < 0.05 *vs.* PP treatment.

**Figure 3:**
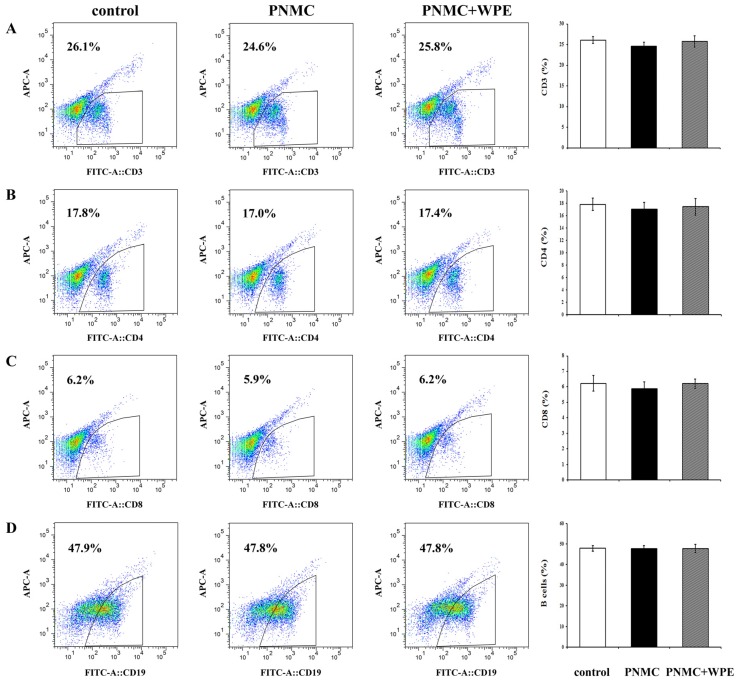
Percentages of various lymphocyte cell types as determined using flow cytometric analysis. (**A**) CD3^+^ T-cells; (**B**) CD4^+^ T-cells; (**C**) CD8^+^ T-cells; and (**D**) B-cells in cells exposed to medium only (control), PNMC (10^−4^ M), or WPE (1.0 μg/mL), together with PNMC for 48 h. Results shown are means ± SD of three separate experiments.

To determine the effects of WPE on splenic T cell populations exposed to PP and PNMC, splenocytes were stained with FITC-labeled antibodies for 48 h and evaluated by flow cytometry. The percentages of CD3+ T and CD8+ T cells were significantly lower in splenocytes exposed to PP relative to controls (Figure 2A,C). Although PP did not have a significant effect on CD4+ T cells, and decreased the cells populations (Figure 2B), with similar results seen for all CD3+, CD4+, and CD8+ T cells exposed to PNMC (Figure 3A–C). However, neither compound appeared to affect the levels of CD19+ B cells (Figure 2D and Figure 3D). Treatment with WPE resulted in higher proportions of CD3+, CD4+, and CD8+ T cells among splenocytes exposed to PP and PNMC (Figure 2 and Figure 3), with overall T cell population numbers similar to those of controls. These results suggest that WPE may restore T cell sub-populations in splenic cells exposed to PP or PNMC.

### 3.3. Treatment with WPE Prevents the Loss of Splenic T Cell Activity

**Figure 4:**
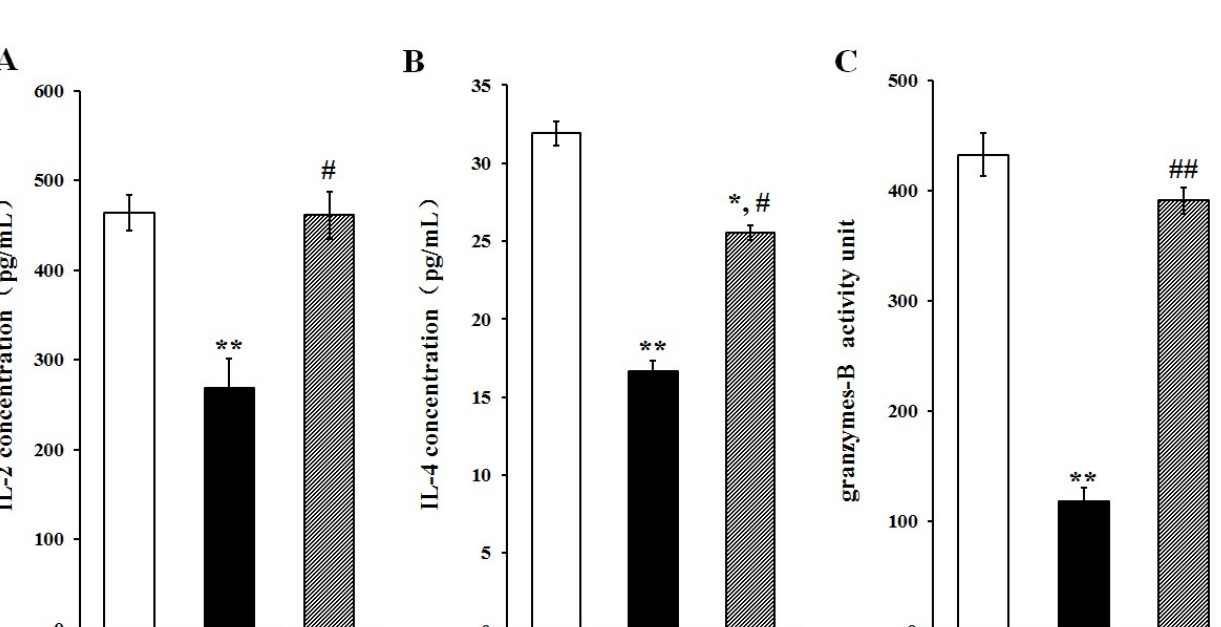
Effect of WPE on select cytokine/granzyme production in splenocytes exposed to PP. Splenocytes were cultured for 48 h in the presence of PP (10^−4^ M) or WPE (1.0 μg/mL) together with PP. Levels of (**A**) IL-2; (**B**) IL-4; and (**C**) granzyme B released into culture media were then measured by ELISA. Results shown are means ± SD of three separate experiments. * *p* < 0.05 or ** *p* < 0.01 *vs.* untreated control; ^#^ *p* < 0.05 or ^##^ *p* < 0.01 *vs.* PP treatment.

**Figure 5:**
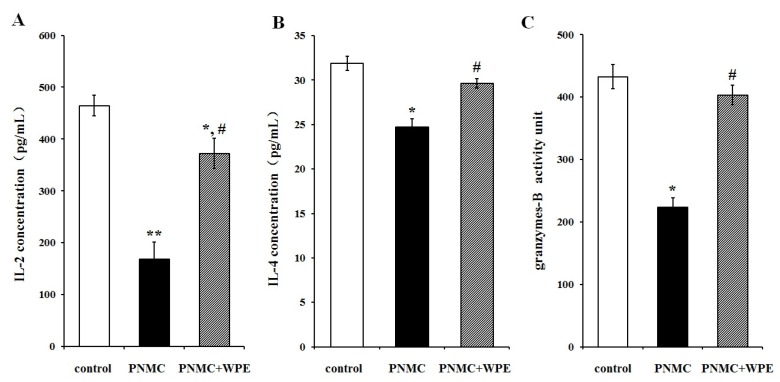
Effects of WPE on select cytokine/granzyme production in splenocytes exposed to PNMC. Splenocytes were cultured for 48 h in the presence of PNMC (10^−4^ M) or WPE (1.0 μg/mL) together with PNMC. Levels of (**A**) IL-2; (**B**) IL-4; and (**C**) granzyme B released into culture media were then measured by ELISA. Results shown are means ± SD of three separate experiments. * *p* < 0.05 or ** *p* < 0.01 *vs.* untreated control; ^#^ *p* < 0.05 *vs.* PNMC treatment.

To determine the effect of WPE on T cell activation, cells were exposed to PP or PNMC for 48 h in the presence or absence of WPE and analyzed for cytokine and granzyme production by ELISA: interleukin (IL)-2, IL-4, and granzyme B were chosen as markers for CD4+ TH1 cells, CD4+ TH2 cells, and CD8+ T cells, respectively. Both PP and PNMC inhibited secretion of IL-2, IL-4, and granzyme B relative to controls (Figure 4 and Figure 5). These effects were significantly attenuated in cells treated with WPE: IL-2 increased from 58% to 99% and 36% to 80% in cells exposed to PP and PNMC, respectively; IL-4 increased from 52% to 80% and 77% to 93%, respectively; and granzyme B increased from 27% to 90% and 52% to 93%, respectively, relative to controls (Figure 4 and Figure 5). These results clearly showed that WPE protected splenocytes from the loss of cytokine/granzyme production in cells exposed to PP and PNMC.

### 3.4. WPE Attenuates PP- and PNMC-Induced Oxidative Damage in Splenocytes

**Figure 6:**
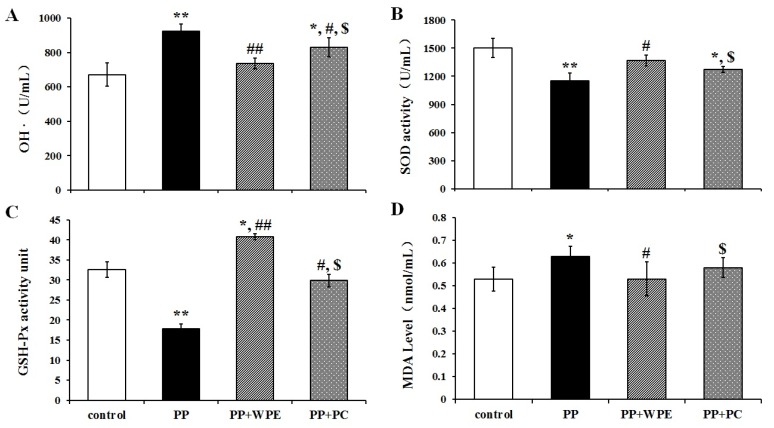
Effects of WPE on levels of ·OH, SOD, GSH-Px, and MDA in splenocytes exposed to PP. Splenocytes were cultured in the presence of PP (10^−4^ M) or WPE (1.0 μg/mL), PC (1.0 μg/mL, together with PP for 48 h. Changes in (**A**) hydroxyl free radical (·OH) activity; (**B**) superoxide dismutase (SOD) activity; (**C**) glutathione peroxidase (GSH-Px) activity; and (**D**) malonaldehyde (MDA) content in the cells were measured using specific assay kits. Results shown are means ± SD of three separate experiments. * *p* < 0.05 or ** *p* < 0.01 *vs.* untreated control; ^#^ *p* < 0.05 or ^##^ *p* < 0.01 *vs.* PP treatment; ^$^ *p* < 0.05 *vs.* WPE (1.0 μg/mL) together with PP (10^−4^ M) treatment.

**Figure 7:**
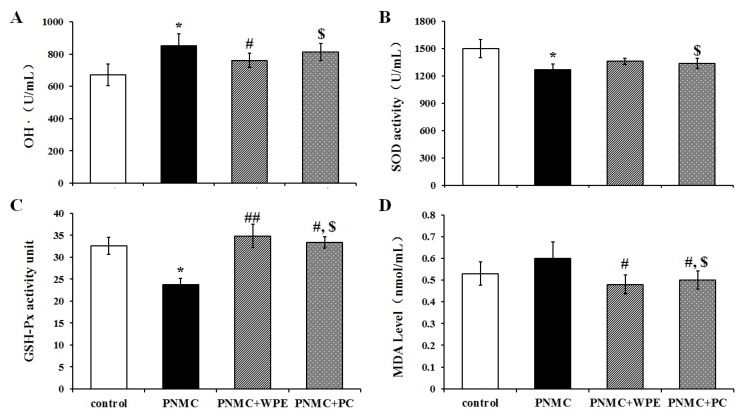
Effects of WPE on levels of ·OH, SOD, GSH-Px, and MDA in splenocytes exposed to PNMC. Splenocytes were cultured in the presence of PNMC (10^−4^ M) or WPE(1.0 μg/mL), PC (1.0 μg/mL) together with PNMC for 48 hr. Changes in (**A**) hydroxyl free radical (·OH) activity; (**B**) superoxide dismutase (SOD) activity; (**C**) glutathione peroxidase (GSH-Px) activity; and (**D**) malonaldehyde (MDA) content in the cells were measured using specific assay kits. Results shown are means ± SD of three separate experiments. * *p* < 0.05 *vs.* untreated control; ^#^ *p* < 0.05 or ^##^ *p* < 0.01 *vs.* PNMC treatment; ^$^ *p* < 0.05 *vs.* WPE (1.0 μg/mL), together with PNMC (10^−4^ M) treatment.

To examine whether WPE can inhibit PP- and PNMC-induced oxidative stress in splenocytes, we examined SOD, GSH-Px, OH, and MDA levels following treatment with PP and PNMC. PP significantly enhanced ·OH and MDA levels in treated cells (Figure 6A,D), while PNMC induced significant increases in ·OH content relative to controls (Figure 7A,D). These levels were significantly attenuated in cells treated with WPE, with decreases in ·OH contents from 923 U/mL to 736 U/mL and 852 U/mL to 761 U/mL in cells treated with PP and PNMC, respectively (Figure 6A and Figure 7A); MDA levels were reduced from 0.63 to 0.53 mmol/mL and 0.60 to 0.48 mmol/mL, respectively (Figure 6D and Figure 7D). In contrast, there were marked decreases in SOD and GSH-Px activity in cells exposed to PP and PNMC. These data are consistent with previous studies that established the effect of PNMC on oxidative stress parameters in several models [22,24]. Treatment with WPE restored SOD activity levels from 76% to 91% and 84% to 90%, relative to controls, in cells exposed to PP and PNMC, respectively (Figure 6B and Figure 7B). Similarly, GSH-Px activity was also increased in WPE-treated cells exposed to PP or PNMC, from 54% or 72% to 125% or 106% of control levels, respectively (Figure 6C and Figure 7C).

Finally, we compared the effects of WPE to those of the polyphenol proanthocyanidin, as this compound has been shown to exhibit strong antioxidant activity [41]. As seen with WPE, proanthocyanidin treatment reduced both ·OH and MDA levels and increased SOD and GSH-Px activities in splenocytes exposed to PP and PNMC; however, the protective effects were lower than those of WPE (Figure 6C and Figure 7C). Taken together, these results indicate that WPE can inhibit both PP- and PNMC-mediated oxidative stress in splenocytes at levels greater than those by proanthocyanidin.

### 3.5. Quantification and Characterization of Phenolic Compounds in WPE

Given the strong antioxidant effects of WPE in splenocytes, we next examined the phenolic constituents of WPE. Total phenolic content was quantified using the Folin–Ciocalteu phenol assay, revealing an average content of 34,800 ± 200 mg gallic acid equivalents (GAE)/100 g. Individual compounds were then identified by LC-MS analyses, with the names and *m*/*z* scores of each compound listed in Table 1. These results are considered tentative. A total of 16 individual phenolic compounds were identified, including ellagitannins (1,2,4,5,7,10–16), quercetin (3,9), valoneic acid dilactone (6), and gallic acid (8). In-depth studies should be performed to further clarify and characterize the phenolic compounds in WPE, and to determine the specific role of each constituent in the protective effect of WPE.

## 4. Discussion

The data presented here show that WPE attenuated PP- and PNMC-mediated toxicity in murine splenocytes, characterized by significant increases in cell viability following treatment with WPE, as well as increases in the proportions of splenic CD3+, CD4+, and CD8+ T cells following exposure to the agents. These increases in T cell populations were accompanied by strong increases in the secretion of IL-2, IL-4, and granzyme B in WPE-treated cells, relative to those exposed to PP or PNMC alone. Examination of ·OH, SOD, GSH-Px, and MDA levels showed that WPE administration significantly inhibited oxidative damage in treated cells. Finally, we determined the total phenolic content of WPE to be 34,800 ± 200 mg GAE/100 g, consisting of at least 16 phenolic compounds based on LC-MS analyses. Taken together, these findings suggest that WPE may provide protection against PP- and PNMC-mediated immunotoxicity in splenocytes by inhibiting oxidative stress.

In recent years, the ability of natural products to both treat and prevent diseases has gained significant attention. Polyphenols have been shown to protect against certain toxicities caused by vehicle emissions. Two compounds, a polyphenol extract of *Ginkgo biloba* and quercetin were shown to protect against endocrine disruptions caused by diesel exhaust [1], while rosmarinic acid significantly attenuated lung injury caused by diesel exhaust [42]. Similarly, *in vitro* treatment of rat primary neurons with partridgeberry polyphenols significantly attenuated β amyloid-induced cell death and membrane damage [43]. Preliminary studies of walnut polyphenols revealed significant anti-oxidative properties [27], limiting oxidative damage induced by CCl_4_ and β amyloid protein [29,31]. In this study, WPE significantly attenuated cytotoxicity caused by PP and PNMC in splenocytes. Furthermore, this protective effect was greater than that seen with two other polyphenols, proanthocyanidin, and quercetin (Figure 1C,D).

Polyphenols may also help maintain proper T cell function. Treatment of normal human peripheral blood lymphocytes with cacao liquor polyphenol *in vitro* regulated T cell proliferation in a concentration-dependent manner [44]. Similarly, the phenolic compounds in *P. glaucum* grains modulated splenic T cell growth in rats [45], while *L. guyonianum* polyphenols are capable of inducing both T and B cell proliferation [46]. In contrast, oligomeric proanthocyanidins from blueberry leaves inhibited the growth of human T cell lymphotropic virus type 1-associated cell lines by inducing apoptosis and cell cycle arrest [47], indicating a more complex regulatory effect for these compounds. Similarly, icariin flavonoid glucoside has been shown to ameliorate autoimmune encephalomyelitis by suppressing the proliferation of T cells and the differentiation of Th1 and Th17 cells among splenocytes and lymph node cells [48]. The results presented here showed that WPE restored the proportion of T cell sub-populations to a level at or near that of controls. This observation is in general agreement with many previous studies suggesting that polyphenols may be useful for maintaining proper T cell function.

Cytokines are essential regulators of both innate and adaptive immune responses, which can be influenced by a variety of dietary antioxidants [49,50,51]. Supplementation of sea bass fed with polyphenols extracted from red grapes increased their production of interferon (IFN)-γ, which may protect against loss of immune function in animals exposed to continuous antigenic pressure by microbes and environmental agents [50]. Ellagic acid and polyphenols present in walnut kernels increased the secretion of IL-2 in peripheral blood mononuclear cells [51], while the expression of IL-3, IL-4, IL-1R2, IL-6R, and IL-7R2 were all upregulated in response to tea polyphenols following alcohol-induced liver injury in rats [52]. Similarly, green tea polyphenols regulated both IFN-γ and TNF-α secretion in colon and lamina propria lymphocytes in a murine model of inflammatory bowel disease [53]. In the present study, treatment of splenic lymphocytes with WPE led to increases in the production of T cell-related effector molecules in cells exposed to PP and PNMC, consistent with previous studies.

In this study, the total phenolic content of WPE was 34,800 ± 200 mg GAE/100 g, which was greater than that seen in other studies (2464–11,500 mg GAE/100 g) [38,54,55]. In total, 16 polyphenolic compounds, including ellagitannins, quercetin, valoneic acid dilactone, and gallic acid, were identified in the extract [37,38,39,40]. Ellagitannins have been reported to stimulate the immune system by activating macrophages and promoting the release of Il-1β [56]. Quercetin has been shown to have a protective effect on immune function by decreasing lymphocyte DNA damage *in vivo* [57]. Gallic acid may inhibit murine leukemia WEHI-3 cells *in vivo* and promote macrophage phagocytosis [58]. In this study, the polyphenolic compounds extracted from WPE, including ellagitannins, quercetin, and gallic acid, had a protective effect against immunotoxicity induced by PP and PNMC in murine splenic lymphocytes.

In recent years, several lines of evidence have shown that dietary polyphenols can protect against oxidative damage caused by a variety of substances. The polyphenolic compounds found in raspberry seeds prevented damage to human peripheral blood lymphocytes by reactive oxygen species [59], while green tea polyphenols protected against nicotine-induced oxidative stress by significantly elevating plasma catalase and SOD activities in male rats [60]. Supplementation with quercetin (1.0 μg/mL) attenuated the toxicity of three nitrophenol components of diesel exhaust by limiting the production of ·OH and MDA, while simultaneously increasing GSH-Px and SOD activities in embryonic chicken testicular cells *in vitro* [22,61,62]. Similar effects on MDA levels and SOD activity were also seen in brain tissue following administration of walnut polyphenols, leading to improvements in both learning and memory functions [63].

The data presented here showed that WPE was able to increase SOD and GSH-Px activities, while decreasing the levels of both ·OH and MDA in splenocytes after exposure to PP and PNMC. SOD and GSHPX activities are associated with cellular enzymatic defense against free radical attacks. The ·OH radical is one of the most toxic and harmful ROS, and MDA is an end-product of lipid peroxidation in cells. Although there was no direct evidence in this study that the protective effect of WPE against PP- or PNMC-induced immunotoxicity was due to its activity against oxidative damage, past findings indicate that certain WPE phenolic compounds are able to protect immune cells from oxidative damage by inhibiting the formation of excessive free radicals.

## 5. Conclusions

Here, we showed that WPE protected against PP- and PNMC-mediated toxicity in splenic lymphocytes, limiting the damaging effects of these compounds on both cell viability and cytokine/granzyme production *in vitro*. The data also support the idea that the protective effect of WPE may at least be partly due to the attenuation of oxidative damage. Further studies will be necessary to identify the active phenolic compounds in WPE and to determine the mechanisms underlying these protective effects.

## Figures and Tables

**Table 1:** Identification of phenolic compounds in WPE using HPLC-ESI-IT-TOF-MS in the negative ion mode.

No.	*t*_R_ (min)	Measured [M − H]^−^(*m*/*z*)	Predicted [M − H]^−^ (*m*/*z*)	λ (nm)	Molecular Formula	Identification	Expressed as	Reference
1	12.663, 12.906	300.9993	300.9990	280	C_14_H_6_O_8_	Ellagic aci	Ellagitannins	[37,38,39]
2	11.946	433.0432	433.0412	280	C_19_H_14_O_12_	Ellagic acid 4-*O*-xyloside	Ellagitannins	[37,38,39]
3	12.507	433.1119	433.0772	280	C_20_H_18_O_11_	Quercetin pentoside isomer	Quercetin	[38]
4	11.946	456.0383	457.0781	280	C_22_H_17_O_11_	Epigallocatechin-3-*O*-gallate	Ellagitannins	[40]
5	13.74	447.0831	447.0938	280	C_21_H_19_O_11_	Kaempferol-3-*O*-glucoside	Ellagitannins	[40]
6	12.507, 12.663, 12.906	469.0492	469.0049	280	C_21_H_10_O_13_	Valoneic acid dilactone	Valoneic acid dilactone	[38]
7	11.946	481.5355	481.0620	280	C_20_H_18_O_14_	2,3-*O-*HHDP-d-glucoside	Ellagitannins	[38]
8	9.828, 10.020, 10.512, 10.784	483.5192	483.0777	280	C_20_H_20_O_14_	Digalloyl-glucose isomer	Gallic acid	[38,39]
9	12.25	615.5905	615.0986	280	C_28_H_24_O_16_	Quercetin galloylhexoside isomer	Quercetin	[38]
10	8.382, 8.751, 9.624	633.0720	633.0720	280	C_27_H_22_O_18_	Strictinin (galloyl-HHDP-glucose)	Ellagitannins	[37,38,39]
11	10.784, 11.136	635.0876	635.0877	280	C_27_H_24_O_18_	Trigalloyl-glucose isomer	Ellagitannins	[38]
12	8.382, 8.751, 9.003	783.0685	783.0681	280	C_34_H_24_O_22_	Pedunculagin (bis-HHDP-glucose)	Ellagitannins	[37,38,39]
13	10.020, 10.236	785.0808	785.0840	280	C_34_H_26_O_22_	Tellimagrandin I isomer(digalloyl-HHDP-glucose)	Ellagitannins	[37,38,39]
14	11.946, 12.250	787.1144	787.0996	280	C_34_H_28_O_22_	Tetragalloyl-glucose	Ellagitannins	[38]
15	10.784, 11.488	933.0316	933.0630	280	C_41_H_26_O_26_	Praecoxin D	Ellagitannins	[37,38,39]
16	11.136	935.0746	935.0786	280	C_41_H_28_O_26_	Casuarinin(Galloyl bis HHDP glucose)	Ellagitannins	[37,38,39]
